# Public health impact of the spread of *Anopheles stephensi* in the WHO Eastern Mediterranean Region countries in Horn of Africa and Yemen: need for integrated vector surveillance and control

**DOI:** 10.1186/s12936-023-04545-y

**Published:** 2023-06-19

**Authors:** Samira M. Al-Eryani, Seth R. Irish, Tamar E. Carter, Audrey Lenhart, Adel Aljasari, Lucia Fernández Montoya, Abdullah A. Awash, Elmonshawe Mohammed, Said Ali, Mohammed A. Esmail, Abdulhafid Hussain, Jamal G. Amran, Samatar Kayad, Mujahid Nouredayem, Mariam A. Adam, Lina Azkoul, Methaq Assada, Yasser A. Baheshm, Walid Eltahir, Yvan J. Hutin

**Affiliations:** 1 Department of Universal Health Coverage/Communicable Diseases Prevention and Control, Eastern Mediterranean Regional Office, World Health Organization, Cairo, Egypt; 2 World Health Organization, Headquarters, 1211 Geneva, Switzerland; 3 Baylor University, Waco, TX USA; 4 Entomology Branch, Division of Parasitic Diseases and Malaria, Center for Global Health, Centers for Disease Control and Prevention, Atlanta, GA USA; 5 World Health Organization, Country Office, Sana’a, Yemen; 6 World Health Organization, Country Office, Sub-Office, Aden, Yemen; 7 World Health Organization, Country Office, Khartoum, Sudan; 8 National Malaria Control Programme, Ministry of Health Development, Hargeisa, Somaliland; 9 National Malaria Control Programme, Ministry of Public Health & Population, Sana’a, Yemen; 10 National Malaria Control Program, Ministry of Health, Garowe, Somalia; 11 World Health Organization, Country Office, Mogadishu, Somalia; 12 National Malaria Control Programme, Ministry of Health, Djibouti, Djibouti; 13 National Malaria Control Programme, Ministry of Public Health & Population, Aden, Yemen; 14 Directorate of the Integrated Vector Management (IVM), Federal Ministry of Health, Khartoum, Sudan

**Keywords:** *Anopheles stephensi*, Invasive vector, Malaria, Vector surveillance, Eastern Mediterranean Region, Breeding sites

## Abstract

**Background:**

*Anopheles stephensi* is an efficient vector of both *Plasmodium falciparum* and *Plasmodium vivax *in South Asia and the Middle East. The spread of *An. stephensi* to countries within the Horn of Africa threatens progress in malaria control in this region as well as the rest of sub-Saharan Africa.

**Methods:**

The available malaria data and the timeline for the detection of *An. stephensi* was reviewed to analyse the role of *An. stephensi* in malaria transmission in Horn of Africa of the Eastern Mediterranean Region (EMR) in Djibouti, Somalia, Sudan and Yemen.

**Results:**

Malaria incidence in Horn of Africa of EMR and Yemen, increased from 41.6 in 2015 to 61.5 cases per 1000 in 2020. The four countries from this region, Djibouti, Somalia, Sudan and Yemen had reported the detection of *An. stephensi* as of 2021. In Djibouti City, following its detection in 2012, the estimated incidence increased from 2.5 cases per 1000 in 2013 to 97.6 cases per 1000 in 2020. However, its contribution to malaria transmission in other major cities and in other countries, is unclear because of other factors, quality of the urban malaria data, human mobility, uncertainty about the actual arrival time of *An. stephensi* and poor entomological surveillance.

**Conclusions:**

While *An. stephensi* may explain a resurgence of malaria in Djibouti, further investigations are needed to understand its interpretation trends in urban malaria across the greater region. More investment for multisectoral approach and integrated surveillance and control should target all vectors particularly malaria and dengue vectors to guide interventions in urban areas.

## Background

The World Health Organization (WHO) estimated that, in 2021, 247 million cases of malaria and 619,000 deaths occurred worldwide, with 95% of cases and 96% of deaths in Africa [1]. In 2016, the WHO member states adopted the WHO Global Technical Strategy (GTS) for Malaria 2016–2030, committing to reduce malaria incidence and mortality by 90% by 2030 [2]. The World Malaria Report 2022 indicated that progress since 2015 has stalled, pointing to the need to analyse obstacles and increase efforts towards control and elimination [1]. The WHO estimated that in 2021, 6.2 million malaria cases occurred in the Eastern Mediterranean Region (EMR), an increase of 44% from 2015 [1]. In EMR, six countries are in the burden reduction phase (Afghanistan, Djibouti, Pakistan, Somalia, Sudan, and Yemen) and two countries are approaching elimination (Islamic Republic of Iran and Saudi Arabia). The remaining 14 countries are preventing malaria re-establishment. Two countries are WHO-certified malaria-free: United Arab Emirates in 2007 and Morocco in 2010 [1]. Sudan accounted for 54% of cases [1]. The Afrotropical *Anopheles arabiensis*, a member of the *Anopheles gambiae* species complex, is a major malaria vector in Djibouti, and the primary vector in Somalia, and Sudan, as well as Saudi Arabia and Yemen in the Arabian Peninsula. Both *Plasmodium falciparum* and *Plasmodium vivax* are transmitted in these countries, with *P. falciparum* contributing to the majority of the cases [1]. *Anopheles arabiensis*, which is mainly a rural malaria vector breeds in a variety of habitats in agricultural areas and in both permanent and transient ground pools, particularly in dry and semi-arid areas [3–7].

*Anopheles stephensi* is an efficient vector of both *P. falciparum* and *P. vivax*. Until 2011, its reported distribution was confined to certain countries in South Asia and large parts of the Arabian Peninsula excluding southwest in Saudi Arabia and Yemen [8–10]. Invasive *An. stephensi* was first reported in Djibouti in 2012 [11]. Since then, its presence has been reported from Ethiopia (2016) [12], Sri Lanka (2017) [13], Sudan (2016) [14] and Somalia (2019) [15, 16]. In 2019, a WHO Vector Alert recognized the threat of emergence and spread of *An. stephensi* outside its native geographical range, calling for vigilance and actions [8]. The presence of *An. stephensi* has since been reported from Nigeria (2020), Yemen (2021), and Kenya (2022) [16]. *Anopheles stephensi* appears to be better adapted in urban and peri-urban areas than *An. arabiensis*. It breeds mainly in water tanks, water storage containers, construction sites, desert coolers, wells and other human-made habitats [10, 17–22]. It is a major malaria vector in urban settings in India that successfully sustains malaria transmission even at low vector densities [23, 24]. Larval control through larviciding and distribution of larvivorous fish are mainly used to interrupt malaria transmission in urban areas in India. The organophosphate insecticide temephos and the bacteria *Bacillus thuringiensis* var israelensis (Bti) are commonly used larvicides [24]. In EMR, there is widespread resistance of *An. stephensi*, to the four major insecticide classes, organochlorines, pyrethroids, organophosphates and carbamates which include commonly used insecticides in the two-core malaria vector control interventions, insecticide-treated nets (ITNs) and indoor residual spraying (IRS) that target the adult stage of the vector [25, 26].

The emergence and spread of *An. stephensi* threatens progress in malaria control in the Horn of Africa and sub-Saharan Africa. Therefore, the data related to the role of *An. stephensi* in malaria transmission in the Eastern Mediterranean Region, specifically in the Horn of Africa and the southwestern part of the Arabian Peninsula was reviewed, including the distribution, genetic diversity and bionomics with focus on the type of vector breeding sites and the insecticide resistance in countries.

## Methods

### Scope and population

The situation of the four countries (Djibouti, Somalia, Sudan and Yemen) in the Eastern Mediterranean Region (EMR) where *An. stephensi* was detected for the first time between 2012 to 2021 was analysed.

### Data sources

#### Epidemiological country reports to the World Health Organization

Two types of country reports to the WHO were examined: the national-level country data on malaria incidence and the sub-national data for the reported malaria cases from major cities. The national-level report data to the WHO on an annual basis through the WHO Integrated data platform (DHIS2) for the World Malaria Report. The sub-national data was obtained from national health facility-based disease surveillance systems in countries, including the national Health Management Information System (HMIS) in Somalia and Sudan and the national electronic Disease Early Warning System (eDEWS) in Yemen.

#### Entomological surveillance reports

Entomological surveillance reports were reviewed, which included detection of *An. stephensi* and detections included in Malaria Threats Map (https://apps.who.int/malaria/maps/threats/), an official WHO digital platform developed to track the spread of this vector. The platform includes detections reported in published scientific literature and detections reported to WHO by countries using a standard spreadsheet template. The template includes the geographical location of the detection, the development stage of the collected mosquitoes (adults or immature stages), the type of larval habitat (collection of immature stages) and the method of mosquito collection. The WHO checks the quality of unpublished country-reported data before uploading it to Malaria Threats Map.

#### Published studies

A literature review was conducted in the search engine PubMed (https://pubmed.ncbi.nlm.nih.gov/) for scientific publications on *An. stephensi* in the Horn of Africa and the Arabian Peninsula. The data for bionomics, insecticide resistance status and genetic diversity of the invasive vector populations was reviewed.

### Data analysis

#### Estimated national malaria incidence in Djibouti, Somalia, Sudan and Yemen

The estimated malaria incidence was plotted for Djibouti, Somalia, Sudan and Yemen from 2010 to 2020 using the population denominator from the World Malaria Report 2021 for estimation of incidence (Annex 5 F). The year of the first reported detection of *An. stephensi* was indicated for each country.

#### Reported sub-national urban malaria incidence

The sub-national malaria incidence was plotted using reported confirmed cases divided by population at risk, over different time periods for the major cities where the *An. stephensi* was detected. These were Bossaso city (2012–2021) in Somalia, Khartoum City (2016–2021) in Sudan and Aden City (2015–2021) in Yemen. For Djibouti, malaria cases are highly concentrated and reported from Djibouti City, therefore the plotted estimated national malaria incidence also represented the urban malaria incidence.

#### Geographical distribution and population structure of *Anopheles stephensi* in Djibouti, Sudan, Somalia, and Yemen

The cumulative number of sites that detected *An. stephensi* was mapped overlaid with population density [27, 28] and created a timeline for reported detection of *An. stephensi* for each country. Previous findings from molecular surveillance and the results of investigations into the genetic diversity of *An. stephensi* were also summarized*.*

### Bionomics and insecticide resistance status

The larval ecology and the insecticide resistance status of *An. stephensi* reported in Djibouti, Somalia, Sudan and Yemen using both unpublished country reports to the WHO and published literature were summarized. The types of larval habitats where studies and countries collected immature stages of *An. stephensi* were listed.

## Results

### Estimated national malaria incidence in Djibouti, Somalia, Sudan and Yemen

**Fig. 1:**
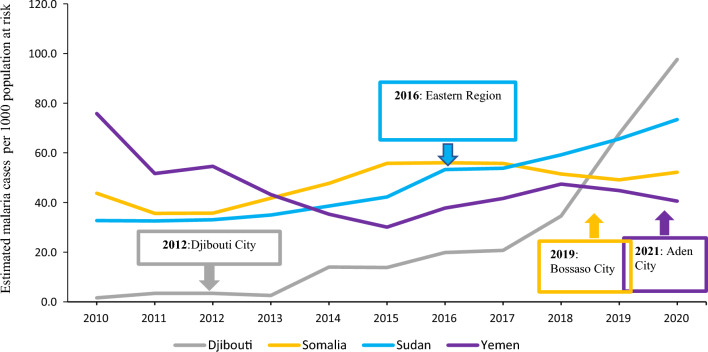
Estimated malaria incidence and timeline of first reported detection of invasive *An. stephensi,* in 4 EMR countries, 2010–2020. Boxes indicate the date of first *An. stephensi* detection in each country

In the subregion of Horn of Africa and Yemen, the estimated incidence of malaria per 1000 population per year decreased from 44.9 per 1000 in 2010 to 41.6 per 1000 in 2015. However, it increased after 2015, reaching 61.5 cases per 1000 population in 2020. As of 2021, four countries of the region reported detection *of An. stephensi*. In Djibouti, where approximately 70% of the population lives in the capital city, the estimated malaria incidence per 1000 population was 1.6 per 1000 in 2010 and remained stable to 3.4 per 1000 until 2012 when *An. stephensi* was detected. Then incidence increased from 2.5 per 1000 in 2013 to 97.6 per 1000 in 2020 (an increase by 39-fold). In the three other countries where *An. stephensi* was detected, the association between detection and incidence at the national level was less clear. In Sudan, the estimated incidence per 1000 population increased regularly from 32.7 per 1000 in 2010 to 73.4 per 1000 in 2020 while *An. stephensi* was detected in 2016 [14]. In Somalia, the estimated incidence per 1000 population increased unevenly from 43.7 per 1000 in 2010 to 52.2 per 1000 in 2020 and *An. stephensi* was detected in 2019 [16]. In Yemen, the incidence per 1000 population decreased from 75.8 in 2010 to 40.6 in 2020, and *An. stephensi* was detected in 2021 [16] (Fig. 1).

### Reported malaria incidence in cities in EMR countries

#### Aden City, Yemen

**Fig. 2:**
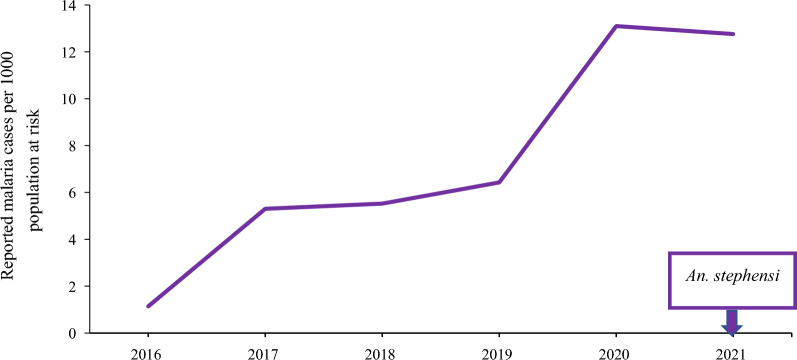
Reported malaria incidence in Aden City, Yemen, 2016–2021. Box indicates the date of first *An. stephensi* detection in the country

The reported malaria incidence in Aden City, Yemen, increased fivefold from 1.1 in 2016 to 5.3 cases per 1000 in 2017. By 2020 and 2021, the reported malaria incidence continued to rise, with 13.1 and 12.8 reported cases per 1000 population, respectively (Fig. 2).

#### Bossaso City, Somalia

**Fig. 3:**
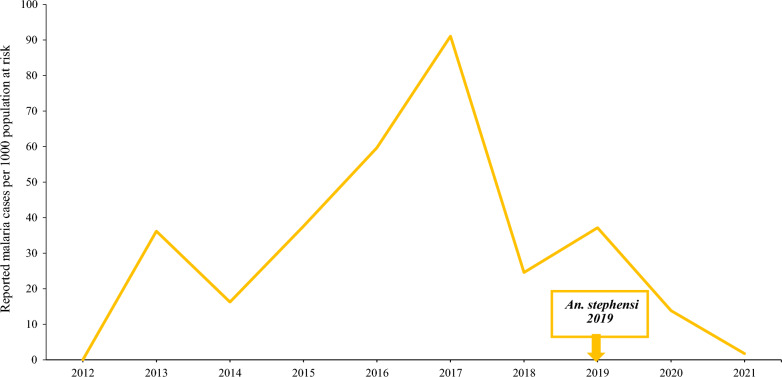
Reported malaria incidence in Bossaso City, Somalia, 2012–2021. Box indicates the date of first *An. stephensi* detection in the country

Malaria incidence in Bossaso City, Somalia was only 0.02 per 1000 population in 2012. The following year, the incidence reached a high of 36.2 per 1000, before dropping to 16.3 per 1000 in 2014. Malaria incidence increased again in 2017 to 91.1 per 1000, dropped to 24.6 per 1000 in 2018 and then rose in 2019 to 37.2 per 1000. However, after 2019, reported malaria cases decreased reaching an incidence of 1.8 per 1000 population in 2021 (Fig. 3).

#### Khartoum City, Sudan

**Fig. 4:**
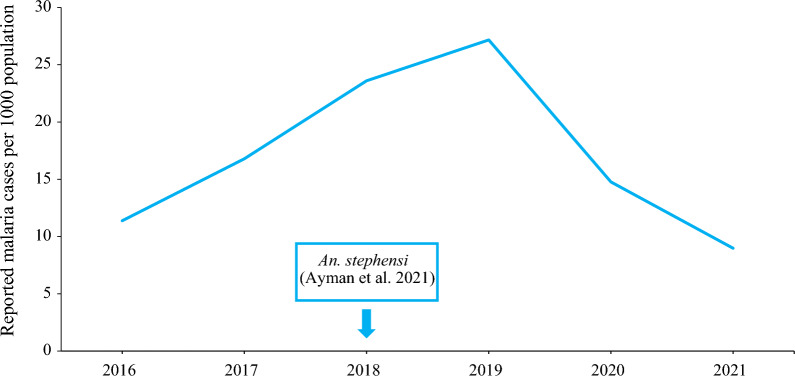
Reported malaria incidence in Khartoum City, Sudan, 2016–2021

Khartoum State contains the largest city of Khartoum, the capital of Sudan. The reported malaria incidence per 1000 population was 11.4 in 2016, with an increase in the following years to reach 27.2 in 2019. In 2020 and 2021, the reported incidence gradually decreased to 9.0 per 1000 population (Fig. 4).

### Geographical distribution of *An. stephensi* in Djibouti, Sudan, Somalia, and Yemen

**Fig. 5:**
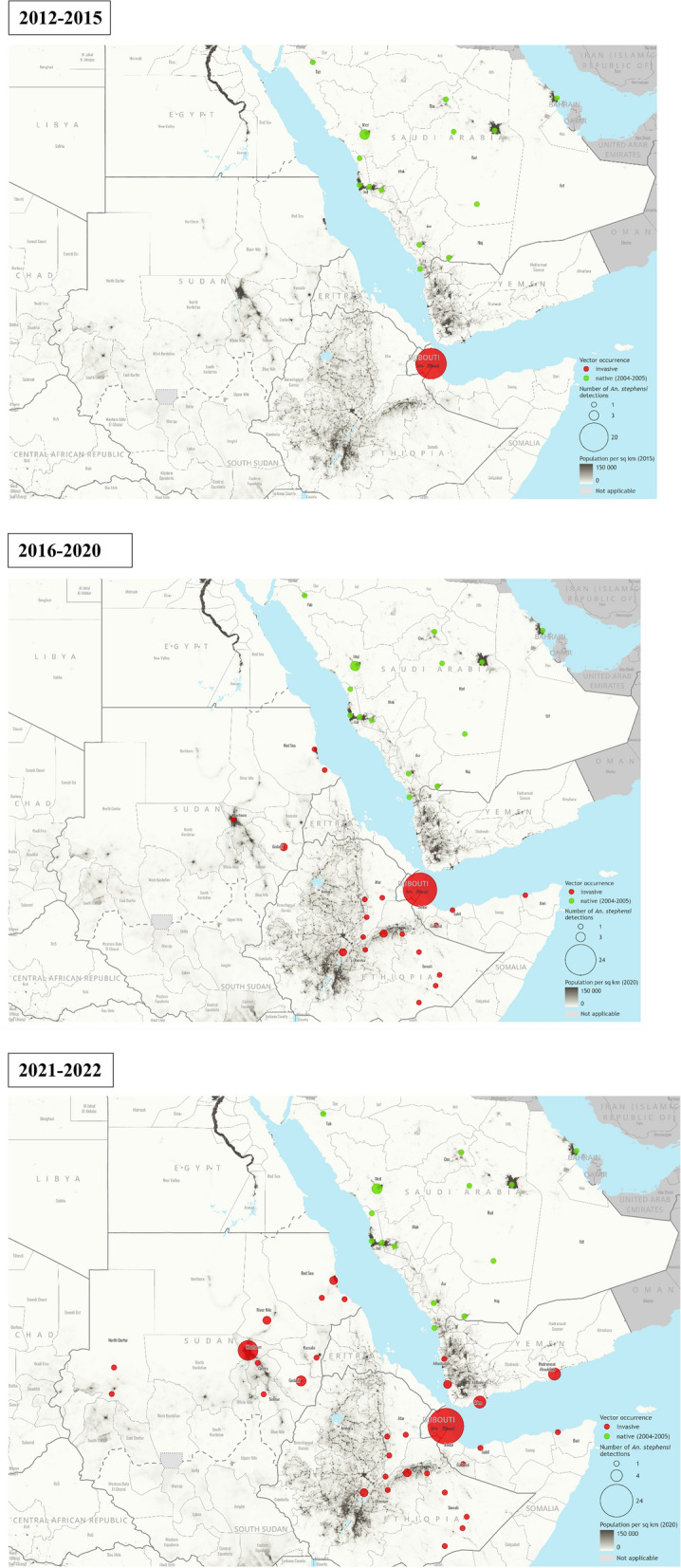
Detections of *An. stephensi* in countries in the Eastern Mediterranean Region in the Horn of Africa and the Arabian Peninsula reported to WHO, overlaid with population density, 2012–2021. Maps credit: WHO GIS Centre for Health

In 2012, Djibouti City reported invasive *An. stephensi* in one site, near the animal export and quarantine station located, approximately 14 km from Djibouti City and 4 km from the Somalian border [11]. Djibouti reported detecting *An. stephensi* in 6 sites in 2013 and 8 sites in 2014 [11] (Fig. 5).

In 2019, Sudan reported *An. stephensi* from 4 sites in collections conducted from 2016 to 2018 [14, 16]: Port Sudan, Tokar, Abu Alnaja and Diam Bakur in Eastern States. A following report of collections in 2018 identified *An. stephensi* in Tuti Island in the capital Khartoum [29]. In 2021–2022, Sudan reported collections from 15 additional sites, which included sites in Khartoum, expanding to a total number of 20 sites, both urban and rural [16, 30].

In 2019, Bossaso City Somalia, was the first Somalian site to report identification of *An. stephensi.* Additional identification followed in 2020 in Hargeisa City, Berbera Port City, and Lawyo Ado, a rural site bordering Djibouti [15] (Fig. 5).

In 2021, Yemen reported the first identification of *An. stephensi*, from Aden City [31] in the south of the country. Additional entomological surveillance in 2021–2022 identified *An. stephensi* in Aden City (3 sites), Lahj governorate (1 site), Al Mukalla City (4 sites) and Broom Mafia (2 sites) in Hadramout governorate and Al Dahi (1 site) and Zabid City (1 site) in Al Hudaydah governorate [16] (Fig. 5).

In all four countries, most of the sites where *An. stephensi* were detected were urban which may be partially due to the fact that this is where national programmes conducted vector surveillance of the invasive *An. stephensi.*

### Genetic diversity of the invasive *An. stephensi*

Genetic diversity studies based on mitochondrial DNA have been conducted for Djibouti, Somalia, and Sudan. Most of these studies utilize a common barcoding gene cytochrome c oxidase subunit 1, *COI*. These studies detected the presence of multiple haplotypes across this region. The populations of *An. stephensi* are genetically similar [15, 32, 33]. The predominant *COI* haplotypes in these countries that were first reported in Ethiopian *An. stephensi* [33] have been reported in South Asia but are absent in most of the long-established *An. stephensi* populations in the Arabian Peninsula. Work on the genetic diversity of new populations in Yemen is ongoing but initial evidence from Aden City indicates the presence of a Horn of Africa haplotype among Yemeni *An. stephensi* [31].

### Bionomics

**Fig. 6:**
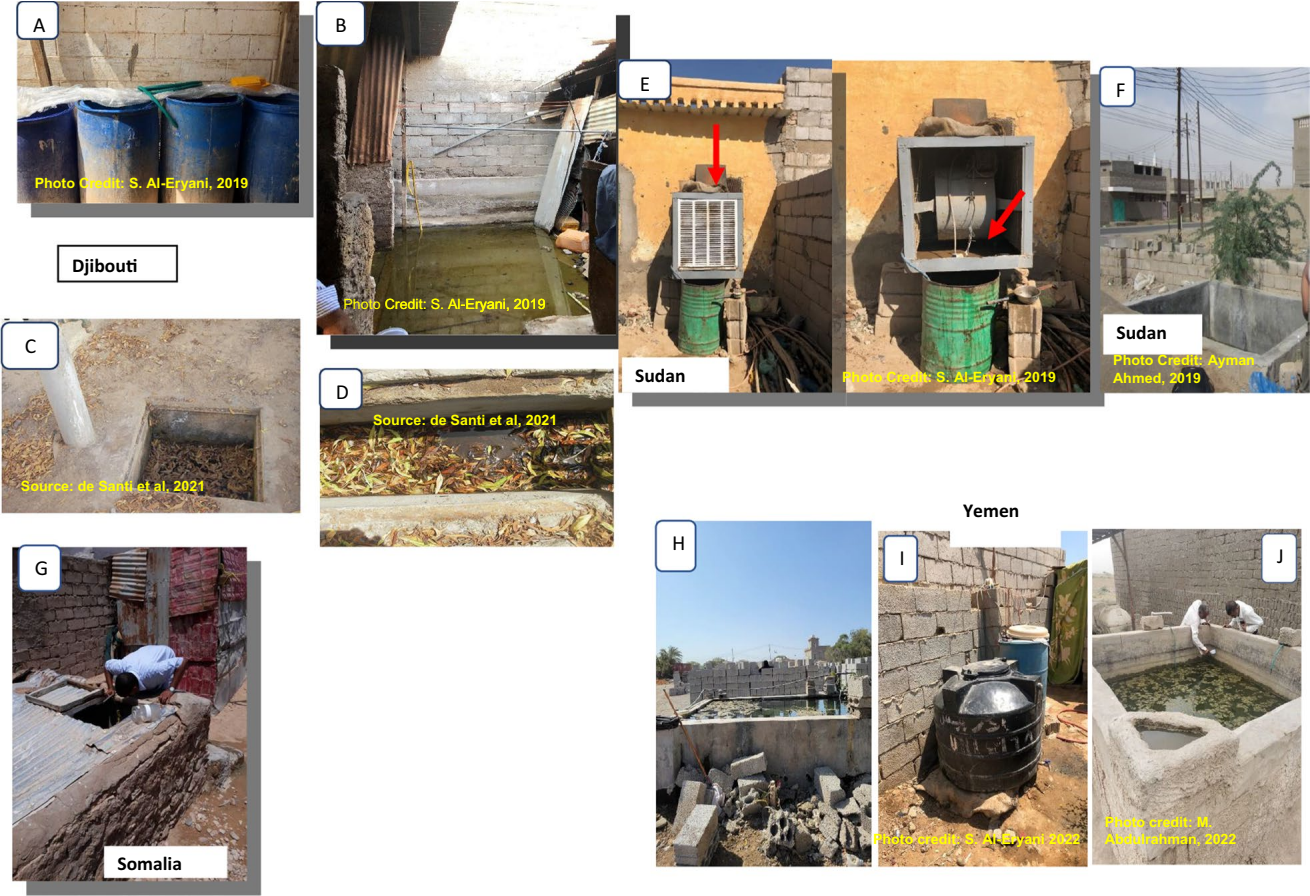
Common breeding sites for invasive *Anopheles stephensi* and *Aedes aegypti* in Djibouti (**A**–**D**), Somalia (**G**), Sudan (**E**, **F**) and Yemen (**H**–**J**), 2019–2022

Available published studies describing the distribution of *An. stephensi* and reports of unpublished entomological surveys conducted by the national malaria control programmes point to common breeding sites of *An. stephensi* across countries (Table 1 and Fig. 6). Similar larval habitats include barrels, household water storage containers, and cement/metal/plastic tanks in Djibouti, Sudan and Yemen. In Djibouti City, in addition to the variety of human-made breeding habitats, water seepage inside houses creates productive breeding sites for both *An. stephensi* and *Aedes aegypti* (author’s field observation (Al-Eryani SM)). In Somalia, *An. stephensi* were collected only from locally made mud/cement water-storage reservoirs ‘Berkads’. Yemen also reported car tyres as *An. stephensi* larval habitats. These breeding sites are also the common breeding sites of *Aedes aegypti*, the primary dengue vector in the region.

**Table 1:** Types of breeding sites from which positive (Presence) *An. stephensi* collections have been made, place and time

Breeding sites where *An. stephensi* has been found	Sudan	Djibouti	Yemen	Somalia
Drum/barrel	Present [30]	Present [32], Identified by the author in 2019	Present [54]	N/A
Bucket/other household water storage	Present [30]	Present [11]	N/A	N/A
Construction water storage reservoir/cement tanks	Present [29, 30]	N/A	Present [54]	N/A
Metal/plastic water tank	Present [29, 30]	Present [32]	N/A	N/A
Berkads	N/A	N/A	N/A	Present [15]
Manhole	Present [29]	Present [32]	N/A	N/A
Water leakage	Present [29, 30]	Present [32]	N/A	N/A
Car wash runoff	N/A	N/A	N/A	N/A
Cemented floor	N/A	N/A	N/A	N/A
Desert cooler	Present Identified by the author in 2019	N/A	N/A	N/A
Ditch	N/A	Present [32]	N/A	N/A
Water seepage inside houses	N/A	Present Identified by the author in 2019	N/A	N/A
Puddle/pond	N/A	N/A	N/A	N/A
Septic tank/wastewater pit	Present [29]	N/A	N/A	N/A
Car tyre	N/A	N/A	Present [31]	N/A
Jerrycan	N/A	N/A	Present [31]	N/A

References cited are presented in brackets

### Insecticide resistance

Few country reports were available on the susceptibility of the invasive *An. stephensi* to insecticides. Phenotypic insecticide resistance to the commonly used public health insecticides was reported in populations of *An. stephensi* in Djibouti and in Yemen. *Anopheles stephensi* populations were resistant to pyrethroids, organophosphates, carbamates and organochlorines (DDT) in Djibouti [34] and pyrethroids and carbamates in Yemen [35]. The presence of the knockdown resistance mutation (*kdr*) associated with resistance to pyrethroids and DDT was detected in specimens from Somalia [15].

## Discussion

Since 2016, malaria progress has plateaued, and the world was off track to achieve the 2020 milestones of WHO’s global malaria strategy [1]. Four of the malaria endemic countries in EMR, Djibouti, Somalia, Sudan and Yemen have reported the detection of invasive *An. stephensi,* a new malaria vector in this part of the world.

In Djibouti City the available data suggests an impact of the invasive *An. stephensi* on malaria transmission in urban areas. The incidence started to increase in 2012 after detection of *An. stephensi*, with an overall increase by 39-fold by 2020. *Anopheles stephensi* may explain the resurgence of malaria in Djibouti City, including the major outbreaks in 2018–2020 [11, 32]. The evidence that associates *An. stephensi* as a cause of urban transmission in Djibouti City includes the detection of *An. stephensi* with *P. falciparum* (3.3%) collected from houses of malaria patients in March 2013 and a second collection of *P. falciparum* positive *An. stephensi* in November 2013 which coincided with the malaria outbreaks in 2013–2014 [11]. Furthermore, entomological data from 2019 reported *An. stephensi* to be the predominant *Anopheles* species breeding in a variety of widespread larval sites in Djibouti City [32].

Unlike in Djibouti, there was no association between malaria incidence and *An. stephensi* detections in Aden, Bossaso and Khartoum. Various causes might have led to the fluctuation of malaria cases reported in these cities. Malaria emerged in Bossaso City in 2013. In 2013 and 2014, long lasting insecticidal nets (LLINs) and indoor residual spraying (IRS), were introduced in Bossaso [36]. These may have led to a decrease in reported malaria cases. In contrast, vector control activities were reduced in Djibouti City during this period [37]. Extreme climatic events in Somalia with heavy rains and floods in Bossaso occurred during 2016, with a major outbreak of dengue and chikungunya that year, followed by an increase of the reported malaria incidence up to a transmission peak in 2017. Concurrent malaria and dengue and chikungunya transmission is not often reported [38]. The epidemiological situation of malaria, dengue or their co-infections is not assessed in these cities that have weak health systems [39]. Other interventions in Bossaso city, including the introduction of active case detection, primaquine treatment for gametocyte carriers, implementation of larval source management such as larvivorous fish and ‘berkad’ modification in targeted areas and a drought since 2020 may have contributed to the reduction of cases from 2017 to 2021.

In Aden City, the upward trend of malaria incidence shares common features with the one in Djibouti City. In Yemen, there is moderate-to-low transmission in southern governorates forming the Aden region (Aden, Lahj, Taiz, Abyan and Al Dhale'e) [40]. During 2018–2020, 41% of the cases were reported from Aden City, accounting for 15% of the total reported malaria cases in Yemen. The increase of reported malaria cases in Aden was observed from 2016, one year following the civil war. The military conflicts in 2015 led to a collapse of the health system in Aden City. The lack of delivery of basic health services, including lack of piped water, influx of internally displaced persons and refugees from neighboring Ethiopia and Somalia contributed to unplanned urbanization. According to the available data, the presence of *An. stephensi* in Yemen was not reported prior to 2021, although it may have indeed been present. Prior to 2021, the focus of vector surveillance in Aden was for the dengue vector, *Ae. aegypti* and there was limited capacity for identification of *An. stephensi*. In 2022, Aden and other coastal cities like Mukalla City in the east of the country, where *An. stephensi* was also detected in 2021, were hosting the recent population displacement waves, both internal due to the ongoing conflict and refugees from the Horn of Africa. In addition, there is a trade route between Bossaso, Somalia and Mukulla Port City, Yemen [41]. These important city ports and trade routes might be used for the movement of *An. stephensi* between the Arabian Peninsula and the Horn of Africa.

Introduction of irrigation schemes in a number of States, which included Khartoum, might have contributed to the increase of the estimated malaria cases from 2016 to 2019. Irrigation schemes led to more intense and perennial malaria transmission in neighbouring countries in Ethiopia [42, 43] and Kenya [44]. The Khartoum Malaria-Free Initiative with larval-source management as the cornerstone of malaria control in Khartoum reduced malaria morbidity and mortality between 2000 and 2011 [45, 46]. However, the initiative was neglected in the following years, highlighted in the malaria programme review in 2018 [46]. Revitalization of the initiative including multisectoral approach for larval source management in Khartoum would be a first step for progress towards elimination. In 2020, the declining trend in malaria was likely due to the disruption of services and lockdown due to COVID-19 Pandemic, which reduced the number of attending patients for seeking health care at the health facilities that included malaria diagnosis. COVID-19 decreased outpatients’ consultation for malaria in Rwanda [47]. Covid-19 reduced in access to services in health facilities in 24 countries in Africa and 7 countries in Asia. Malaria diagnosis decreased 56% and 17% in the countries in Asia and Africa, respectively [48].

In the absence of more complete longitudinal data from all sites, genetic data has helped fill the knowledge gap on the origin of *An. stephensi* in the EMR. While genomic investigations are still underway, some clues have been derived from mitochondrial data generated to confirm species.

The work completed in Djibouti, Sudan, and Somalia illustrated the usefulness of the population genetic and phylogenetic analysis of *An. stephensi* populations to narrow the region of origin of the invasive species. The populations of *An. stephensi* are genetically similar [15, 32, 33] which suggests a common origin. On-going genome-wide analyses on *An. stephensi* in the EMR will provide more precise information about the connectivity between *An. stephensi* populations, the potential mode of introduction, and the environmental factors that influence its spread and adaptation. Genomic data will be particularly critical to establish the temporal relationship between *An. stephensi* populations in the Horn of Africa and those detected in Yemen that have yet to be resolved with single gene analysis. The genetic profiles of *An. stephensi* populations generated so far provide a helpful baseline to revisit with follow-up investigations.

The review of the larval habitat data on invasive *An. stephensi* populations pointed to characteristics shared with *Ae. aegypti* [11, 32, 49, 50]. The habitats of invasive *An. stephensi* were mainly in human-made containers, characteristic of native *An. stephensi* in India [20, 51]. In Djibouti City, water seepage inside houses also created productive breeding sites for both *An. stephensi* and *Ae. aegypti* (Al-Eryani, pers. commun.). Similarly, invasive *Ae. aegypti* was collected from typical breeding sites of *An. stephensi* in Iran [52, 53]. While *An. stephensi* was found in several different types of breeding sites in Djibouti [11, 32], Sudan [29, 30] and Yemen [54], only one type of locally human-made container (berkads) was reported as habitats for *An. stephensi* immatures collected in Somalia [15]. In neighbouring Ethiopia, breeding sites reported [12, 55, 56], included berkads and car tyres as in Somalia and Yemen, respectively. Car tyres provide ideal breeding sites for the dengue vector, *Ae. aegypti*. Aden City is one of the cities in Yemen reporting the highest number of dengue and chikungunya cases with frequent outbreaks [57]. *Anopheles stephensi* was coincidently detected for the first time during surveys for the control of *Ae. aegypti* [31]. The different types of breeding sites reported may reflect behavioural differences in *An. stephensi* between sites, differences in the type of breeding sites available at the sites, or the survey methods used.

Available data reported resistance in *An. stephensi* to pyrethroids, organochlorines, organophosphates and carbamates in sites where it has been monitored in Djibouti [34] and Yemen [35]. This is also consistent with the data reported in Ethiopia for 2016–2021 [50, 56] suggesting that *An. stephensi* is resistant to multiple classes of insecticides and to the data reported from other countries in Asia and the Arabian Peninsula [26]. The evidence of resistance to multiple insecticides highlights the need for alternative strategies involving new chemicals or non-chemical-based vector control.

The detection of *An. stephensi* in the geographic locations within these countries of the Horn of Africa, point to a risk of further expansion of *An. stephensi* across Africa, as evidenced by recent findings in Nigeria and Kenya [16]. Predictive maps showing suitable habitat for *An. stephensi* outside its native geographical range suggest that it will continue to spread, potentially exposing at least 126 million additional people at risk of malaria [58]. The WHO continues to track the vector in the WHO Malaria Threats Map, which was set up for this purpose and countries are encouraged to report detections as soon as they are confirmed [16].

There are several limitations to this review. First, data availability, analysis and interpretation of urban malaria data is often of limited quality. The limits of the “urban” area influence the data to determine the actual spread and distribution of *An. stephensi*. Mobility of human populations can complicate the establishment of an association between cases of malaria reported in urban areas and urban transmission. Second, there are also important challenges in understanding the spread of *An. stephensi*. Tracking species invasions is most accurately done when consistent surveillance systems have been in place. In the case of *An. stephensi*, surveillance methods and frequency varied from place to place, with an emphasis on urban and semi-urban areas. Habitats of *An. stephensi* in rural areas may differ from those in urban settings. The detection of *An. stephensi* in a city does not provide much information about when the invasion took place unless there has been rigorous and regular entomological monitoring. This is unfortunately not the case for the cities described here. In some cases, the first *An. stephensi* reports were often the first published report of any mosquito for that site [11, 15]. Thus, a detection of *An. stephensi* in 2019 in Bossaso Somalia, may not necessarily mean that *An. stephensi* first arrived in 2019. Similarly, even the first report from Djibouti does not mean that this port city was the site of introduction. Third, reports of positive detections of *An. stephensi* could constitute a publication bias. Knowing where *An. stephensi* has been looked for but not detected is also essential to understand conditions that prevent establishment of this invasive vector. Systematic data are needed to create a forecasting system that can predict, with confidence, the presence of *An. stephensi* in other areas. Finally, the insecticide resistance status, infectivity and behavior of invasive *An. stephensi* are not well understood. Important gaps remain to fully incriminate *An. stephensi* as an efficient malaria vector in these countries.

## Conclusion

This analysis leads to four major points of conclusion. First, in Djibouti *An. stephensi* is well established and has likely been the driving force for the explosive increase in urban malaria. Second, outside of Djibouti, the impact of invasive *An. stephensi* on malaria trends remains unclear. Third, *An. stephensi* is resistant to many insecticides commonly used in public health, and this poses challenges to effective vector control options. Fourth, breeding sites for *An. stephensi* are often typical habitats for *Ae. aegypti* and the two vector species are frequently found together.

Based on the results of the review, a way forward can be proposed. The control of *An. stephensi* and *Ae. aegypti* should be aligned with the Global Vector Control Response (GVCR) 2017–2030 and EMR Regional plan for implementation of the GVCR 2019–2023, which provide strategic guidance to countries and development partners to strengthen vector control worldwide through increased capacity, improved surveillance, better national and international coordination and integrated action across sectors and diseases [59, 60]. Further to the regional advocacy, recent literature also emphasized an integrated vector control approach [61, 62].

More specifically, routine surveillance for *An. stephensi* is needed where it is currently reported and in sites at high risk of invasion. Research should better characterize the effectiveness of the current malaria vector control interventions, including novel approaches, on the invasive vector. Though molecular surveillance has been implemented in most countries at the point of initial detection, support for continued monitoring is necessary. Continued molecular surveillance of the population structure will be particularly useful for measuring the success of interventions such as larvicidal treatments. Further investigations are needed to understand the link between *An. stephensi* and urban malaria in new areas, particularly in the Afrotropical settings invaded by *An. stephensi.* Finally, available resources need to be used for integrated vector surveillance and control that can target urban disease vectors with intersectoral collaboration and community engagement. To adequately address the growing threat of invasive *An. stephensi* regular consistent monitoring and timely sharing of information can inform mitigation and containment efforts and aid in decreasing the risk for increased malaria in urban settings.

## Data Availability

All available data are included in the manuscript and from publicaly available sources.
